# Organ Size and Metabolic Rate Co‐Vary with Dimensions of the Obstetric Pelvis: The Metabolic Coordination of Childbirth Hypothesis

**DOI:** 10.1002/ajpa.70342

**Published:** 2026-09-03

**Authors:** Jonathan C. K. Wells, Meghan Shirley Bezerra, Rasmus Wibaek, Owen J. Arthurs, Kiran K. Seunarine, Simon Eaton, Chris A. Clark

**Affiliations:** ^1^ UCL Great Ormond Street Institute of Child Health London UK; ^2^ Children's Hospital of Philadelphia Philadelphia Pennsylvania USA; ^3^ Steno Diabetes Center Herlev, Copenhagen Denmark; ^4^ Department of Radiology Great Ormond Street Hospital London UK

**Keywords:** growth, muscle mass, obstetric pelvis, obstetrical dilemma, organ mass

## Abstract

**Objectives:**

Childbirth complications occur if fetal size is excessive for the maternal birth canal. We hypothesized that young women's metabolic traits (organ and muscle mass, resting energy expenditure (REE)) known to promote fetal growth would correlate with dimensions of the birth canal, and that some of this association would be explained by growth markers (tibia length, height).

**Materials and Methods:**

We analyzed cross‐sectional data on anthropometry, organ mass (magnetic resonance imaging (MRI)), skeletal muscle (dual‐energy X‐ray absorptiometry), fat mass (4‐component model), pelvic dimensions (MRI), and REE (indirect calorimetry) in 68 nulliparous women aged 20–28 years of South Asian ancestry living in the UK. Principal component analysis (PCA) was used to summarize variability in organ/muscle mass and pelvic dimensions (PCA_pelvis_). Associations between metabolic and pelvic traits were assessed using Pearson correlations, and partial correlations adjusting for height or tibia length.

**Results:**

Mean height was 161 cm and body mass index 22.3 kg/m^2^. There were positive correlations between REE, organ/muscle mass, head girth and the first PCA_pelvis_ score, which were partly explained by variability in tibia and height. Fat mass was associated with REE and organ/muscle mass but not pelvic dimensions.

**Discussion:**

Correlations between metabolic and pelvic traits indicate a degree of coordination: women with greater metabolic capacity to promote fetal growth also have larger pelvic dimensions, partly explained by linear growth patterns. However, body fat in nulliparous women correlates with REE but not pelvic dimensions; hence high adiposity could increase risk of childbirth complications when these women give birth.

## Graphical abstract

Among South Asian women, those with larger organ mass and energy expenditure, indicating greater capacity to promote fetal growth, had larger pelvic dimensions, partly explained by linear growth patterns. However, body fat correlated with energy expenditure but not pelvic dimensions, helping explain why high adiposity increases risk of obstructed labour.

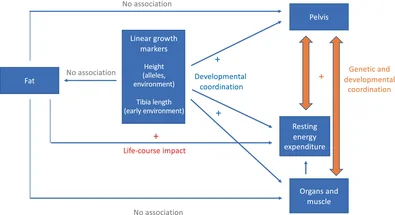

## 1 Introduction

The first evolutionary perspective on human childbirth focused at the level of the species. Washburn proposed that long‐term antagonistic selective pressures had acted on the dimensions of the maternal pelvis and the neonatal head, favoring bipedal locomotion and encephalisation, respectively. The combination of a large‐brained baby and a smaller birth canal was proposed to make childbirth more challenging in *Homo sapiens* , resolved by the ‘relatively early’ delivery of human offspring to reduce neonatal dimensions (Washburn 1960). This perspective stimulated research profiling the complexity of human childbirth, involving rotational delivery and the near‐universal role of social help (Rosenberg and Trevathan 2002; Trevathan 2011). Recently, Washburn's hypothesis has been revisited and critiqued (Dunsworth et al. 2012; Wells et al. 2012; Haeusler et al. 2021; Stone 2016), but the notion that the human pelvis is subject to contrasting selective pressures and is characterized by functional trade‐offs remains valid (Grunstra et al. 2023; Stansfield et al. 2021). For example, a large study of the UK Biobank found that dimensions of the birth canal had moderate coefficients of heritability (25%–40%), and that wider birth canals were associated with reduced walking pace and greater risk of hip osteo‐arthritis, whereas narrower birth canals were associated with reduced risk of pelvic floor disorders, but increased risk of emergency C‐section and obstructed labour (Xu et al. 2025). Other studies link the wider pelvises of women relative to men with an increased risk of knee injury (Hirst et al. 2007).

Evolutionary perspectives are also relevant at the individual level, where childbirth can be considered a coordination problem (Wells 2015, 2017; Mitteroecker et al. 2016). Obstructed labour occurs when the magnitude of fetal growth exceeds the capacity of the maternal birth canal. To reduce this risk, selection may have favored the genetic coordination of maternal and neonatal traits. For example, women with large heads (likely to produce neonates with the same characteristic) have larger pelvic dimensions (Fischer and Mitteroecker 2015; Greulich et al. 1939), while pelvic dimensions also show correlations with offspring birthweight (Wells et al. 2017). However, the metabolic basis of such coordination remains poorly understood.

The primary factor shaping mammalian fetal growth is maternal metabolism and its organ determinants (Martin 1983; Martin and MacLarnon 1985). In humans, maternal fat‐free mass is a strong determinant of offspring birthweight, whereas within the normal range, fat mass shows a weak or null correlation (Wells 2018). Within fat‐free mass, skeletal muscle represents the largest component, but has a relatively low mass‐specific resting metabolic rate (Wang et al. 2010; Shirley et al. 2018). Other organs are smaller in absolute size but have much higher mass‐specific resting metabolic rates (Wang et al. 2010; Shirley et al. 2018). The absolute and relative size of the different organs and tissues thus determines overall resting metabolic rate (Wang et al. 2010; Shirley et al. 2018). Importantly, adipose tissue also has metabolic costs, and being a relatively large tissue it contributes to variability in resting metabolic rate (Garby et al. 1988). However, adiposity is also variable through the life‐course, and within individual women it varies in association with factors such as age, diet, smoking, physical activity and reproductive history (Sowers et al. 1996; Fung et al. 2015; Ramires et al. 2015; Lassek and Gaulin 2006).

Recently, we demonstrated in a cohort of nulliparous South Asian women living in the UK that both organ and skeletal muscle mass (Shirley et al. 2022) and the dimensions of the obstetric pelvis (Shirley et al. 2020) are associated with tibia length, a sensitive marker of environmental conditions experienced in early life (Gunnell et al. 1998). Considered separately, this suggests that poor early growth may constrain both the dimensions of the birth canal and the metabolic tissues that promote fetal growth. In contrast, body fat was not correlated with tibia length (Shirley et al. 2022).

**FIGURE 1:**
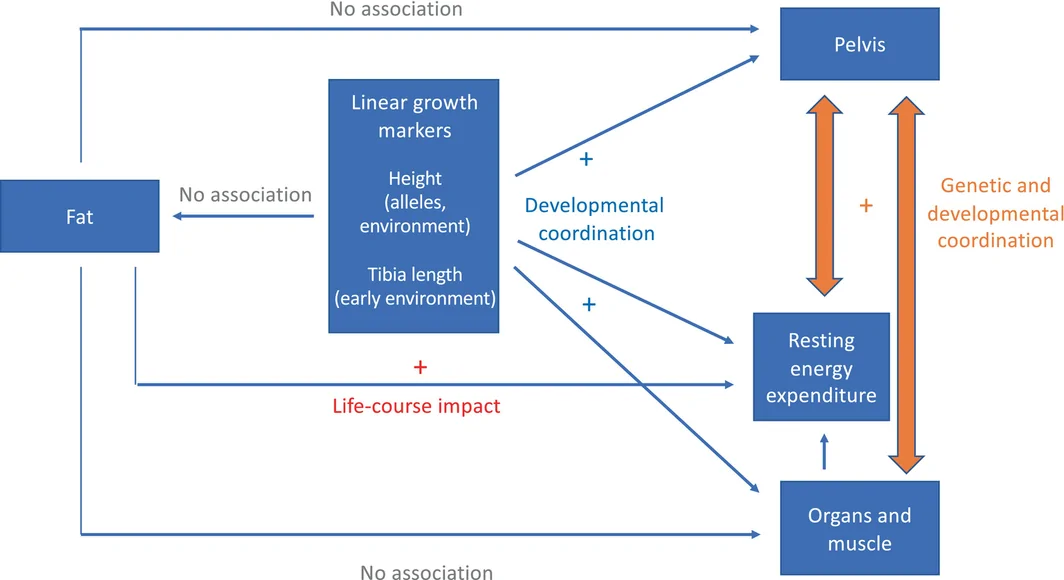
Conceptual diagram illustrating associations between growth and body composition traits with pelvic dimensions, and the different mechanisms through which the association might develop. Larger organ size is expected to drive greater resting energy expenditure. A correlation between organ size and pelvic dimensions is expected, which could reflect common impacts of total growth, proxied by height, or more specifically by growth in early life, proxied by tibia length. These associations between growth, organs and pelvis could in turn involve both genetic drivers and life‐course environmental exposures. Body fat mass is expected to correlate with organ mass and resting energy expenditure, but not with early growth or pelvic dimensions.

Here, in the same sample of women, we analyze the direct correlations between metabolic traits and dimensions of the pelvis. Figure 1 illustrates the expected associations, based both on the literature and on the logic of coordination, which refers to the expectation that natural selection should favor consistency across the metabolic and skeletal dimensions of individual women, to minimize the risk that the capacity to deliver the baby is inadequate, relative to the capacity to promote fetal growth (Wells 2015; Wells et al. 2024). Our objectives were to test the following hypotheses:

- That women with larger organ and skeletal muscle mass, potentially able to support greater fetal growth, would also have larger pelvic dimensions
- That these correlations would partly be explained by the common associations of organ/muscle mass and pelvic dimensions with variability in linear growth, proxied by height and tibia length
- That fat mass, exposed to a range of ecological factors throughout the life‐course, would not correlate with pelvic dimensions

Our study was originally set up to explore brain–body trade‐offs using a life‐history framework, as described previously (Shirley Bezerra et al. 2024). As part of that approach, we quantified the resting metabolic rate of specific organs and tissues (Shirley et al. 2018). A secondary aim was to explore variability in the pelvis and its correlates (Shirley et al. 2020).

Our choice to focus on South Asian women was guided by the fact that populations from this region are among the shortest worldwide (NCD Risk Factor Collaboration 2016) due to a long‐term secular decline in height (Pomeroy et al. 2019). They also have high rates of low birth weight (Wells, Pomeroy, and Stock 2021), an issue of major public health importance which indicates that the constraint of growth begins in utero. An analysis of the skeletal record indicates that thin physique has characterized this geographical region for several millennia (Pomeroy et al. 2019), and in absolute terms, both women's pelvic dimensions (Pan 1929) and adult organ masses are smaller in contemporary South Asian compared to European populations (Kohli and Aggarwal 2006; Wells et al. 2016). The difference in organ size persists after adjusting for height (Wells et al. 2016), however the scenario for pelvic dimensions is unknown. Since selective pressures appear to have favored reduced size in a range of morphological and anatomical traits, we expected that alongside any trade‐offs between such traits, there could a degree of coordination across the physiological and anatomical traits that relate to childbirth.

## 2 Methods

The study inclusion criteria comprised South Asian (Indian, Pakistani, Bangladeshi, Sri Lankan) ancestry, being aged 20–28 years, healthy, nulliparous, term‐born and having body mass index (BMI) in the range 17–28 kg/m^2^. Ethnicity was based on self‐identification, and confirmed by maternal and paternal grandparents being of the same self‐identified ethnic background. The age range was chosen to avoid phenotypic variability associated with pubertal growth or ageing.

Individuals were excluded if they reported health conditions with the potential to affect growth or metabolism; smoking or excessive alcohol use; or contraindications for magnetic resonance imaging (MRI). The BMI criteria were used to exclude women outside the BMI‐defined ‘normative’ range, tailored to the ethnicity of the population (WHO Expert Consultation 2004).

Data were collected from March 2015 to May 2016 at UCL Great Ormond Street Institute of Child Health (GOS‐ICH), London, UK. Ethical approval was granted by the London—Camden and Kings Cross NHS Research Ethics Committee of the Health Research Authority on 15 October 2014 (REC reference: 14/LO/1684). This committee conforms with the Declaration of Helsinki, and additional UK ethical criteria (https://www.hra.nhs.uk/about‐us/what‐we‐do/our‐role‐protecting‐research‐participants/). All participants provided written informed consent prior to their participation in the study. The study was granted approval by the Joint Research and Development Office of UCL GOSICH and Great Ormond Street Hospital on 21 November 2014 (R&D reference: 14NT03). Completed risk assessments for the study were approved by the Head of Laboratory Management and Safety on 24 December 2014.

Recruitment was carried out in London, mainly around UCL, although study details were also circulated at the University of Cambridge. Methods of recruitment included posters in university buildings, circulating study details via email to staff and students of GOSICH, and to UCL societies, and online student newsletters (UCL, London School of Economics, UCL Union). MSB also attended a Sikh Gurdwara (temple) in London to connect with potential participants.

### 2.1 Anthropometry

All anthropometric measurements were taken in duplicate. Weight was measured to the nearest 0.01 kg. Height was measured to the nearest 0.1 cm using a wall‐mounted stadiometer (Holtain, Dyfed, UK), with participants standing barefoot. Large sliding calipers (Harpenden, UK) were used to measure, to the nearest millimeter, tibia length as the distance from the medial tibial plateau to the inferior edge of the medial malleolus on the left leg only, following standard protocols (Pomeroy et al. 2012). Head circumference was measured to the nearest 0.1 cm with a non‐stretch, flexible measuring tape.

### 2.2 Skeletal Muscle Mass

Dual‐energy X‐ray absorptiometry (DXA; Lunar Prodigy, GE Medical Systems, Madison, WI, USA) was used to estimate appendicular (limb) muscle mass. Participants underwent a scan lasting approximately 5 min, with tissue masses obtained directly from the DXA system software (Encore, v14.10.022). The basis for using these data as a measure of whole‐body skeletal muscle mass has been described previously (Kim et al. 2002).

### 2.3 Fat Mass

Fat mass was calculated using the 4‐component model, as described in detail previously (Wells et al. 2015). Briefly, total body water was obtained by deuterium dilution, body volume was measured by air‐displacement plethysmography, and total bone mineral content was obtained from the DXA scan. Combined with data on weight, fat mass was calculated using the following equation:

$$\begin{array}{c} \mathrm{Fat}\;\text{mass}\;\left(\mathrm{kg}\right)=\left(2.747\times \text{body volume}\right)–\left(0.71\times \text{total body water}\right)\\ \;+\left(1.46\times \text{bone mineral content}\right)–\left(2.05\times \text{weight}\right) \end{array}$$

### 2.4 Resting Energy Expenditure

Whole‐body resting energy expenditure (given our protocol, this is the same as basal metabolic rate) was measured in a post‐absorptive state in a thermoneutral environment using a Deltatrac II indirect calorimeter (Datex‐Engstrom Corp, Helsinki, Finland). Participants were not measured at a specified point in their menstrual cycle. However, day‐of‐cycle at data collection (estimated using subject‐reported information on recent menstruation and general cycle length) was found not to be associated with measured resting energy expenditure, and thus rejected as a potential confounder.

Following gas calibration of the instrument, O_2_ consumption and CO_2_ production were assessed continuously for 25 min as participants rested supine on a couch under a ventilated plastic canopy. Using these data, resting energy expenditure was calculated in kcal/24 h units using the equation of Weir (Weir 1949). The measurement could not be scheduled in two participants.

### 2.5 MRI Acquisition

High‐resolution 3D imaging of the brain, chest, abdomen and pelvis was undertaken using a 3 T Siemens Magnetom Prisma scanner (Siemens, Erlangen, Germany). The following were acquired: a T_1_‐weighted MPRAGE sequence for brain volume (TR = 2000 ms, TE = 2.74 ms, flip angle = 8°, voxel size = 1.0 × 1.0 × 1.0 mm isotropic, slices = 240, duration = 5 min); a T_2_‐weighted, turbo spin echo SPACE sequence for the abdomen (TR = 2000 ms, TE = 220 ms, flip angle = variable, voxel size = 1.5 × 1.5 × 1.5 mm isotropic, slices = 144, duration = 7 min); a T_2_‐weighted TrueFISP sequence with breath‐hold for the chest (TR = 475 ms, TE = 1.53 ms, flip angle = 47°, voxel size = 1.5 × 1.5 × 4.0 mm, gap = 0, slices = 42, duration = 20 s); and for the pelvis, a volumetric 3D T_2_‐weighted acquisition using 144 contiguous coronal slices (TR 15.5 ms, TE 5.1 ms, flip angle = 25°, voxel size = 1.2 × 1.2 × 1.2 mm, 1 average, duration = 5 min).

### 2.6 Brain and Body Organ Volume

To derive brain volume (i.e., summed cerebral and cerebellar gray and white matter, and subcortical structures including the amygdala, hippocampus, pallidum, thalamus and striatum), T_1_‐weighted MR images were processed and segmented with FreeSurfer (v5.3) and FIRST (FMRIB Software Library v5.0), as described in detail elsewhere (Fischl et al. 2002, 2004; Patenaude et al. 2011).

The heart, kidneys and liver were manually segmented by a single observer (MSB) from raw MRI data using the open‐source OsiriX DICOM viewer (v8.5) (Rosset et al. 2004). Regions of interest were drawn around the organs of interest in contiguous image slices in each subject dataset. The software automatically calculated organ volume by summing the voxels in the regions of interest and multiplying by the slice thickness. Duplicate organ volumes were derived on different days and averaged. The technical error of measurement for the duplicate measures was 1.9% for the heart, 1.1% for the left kidney, 0.7% for the right kidney, and 0.7% for the liver. These values are considered good quality for body composition outcomes (Ulijaszek and Kerr 1999). Heart volume was missing for 3 participants. The organ volumes were converted to mass using the following published density values in g/cm^3^: 1.036 (brain); 1.060 (heart and liver); 1.054 (spleen); and 1.050 (kidneys) (Duck 1990).

### 2.7 Pelvic Dimensions

Six pelvic dimensions (bi‐iliac breadth, bi‐acetabular breadth, anteroposterior dimensions of the inlet and outlet, inter‐spinous diameter, and inter‐tuberous diameter) were measured using the OsiriX DICOM viewer (Rosset et al. 2004). Straight lines were drawn between anatomical landmarks for each of the six pelvic outcomes as described previously (Shirley et al. 2020). Duplicate measurements were taken on different days and averaged. Pelvic scans were not conducted in two participants. In addition, a small number of measurements were considered unreliable due to reduced image quality, with the pelvic outlet particularly affected. This led to missing data as follows: inlet and inter‐tuberous (*n* = 1), inter‐spinous (*n* = 2) and outlet (*n* = 11).

### 2.8 Statistics

All available data were used in analyzes. Given the large number of individual traits measured, principal component analysis (PCA) was used to extract composite markers of (a) organ/tissue mass and (b) pelvic dimensions. This statistical approach reduces large datasets of inter‐correlated variables to a smaller set of uncorrelated variables (principal components), while retaining maximum variance and information. We used Oblimin (oblique) rotation, which unlike orthogonal rotation allows the principal components to be correlated. This option was considered biologically more realistic for our analysis, given that different sets of traits might demonstrate positive associations or trade‐offs rather than being entirely uncorrelated.

The first PCA model entered data on the brain, heart, liver, kidneys, spleen and muscle. The second PC model entered data on 5 pelvic dimensions, namely bi‐iliac, bi‐acetabular, inlet, inter‐spinous, and inter‐tuberous dimensions. The outlet was excluded due to the 11 missing data points.

To assess associations between metabolic and anatomical traits (PCA scores for organs, brain and pelvis, fat mass, along with resting energy expenditure and head circumference), Pearson correlations were used. Scatter plots were used to visualize associations. Further partial correlations were calculated, first adjusting for tibia length, and then for total height, and visualized using forest plots. These partial correlations were intended to understand to what extent correlations between metabolic and pelvic traits were explained by their co‐variance with markers of growth. We used height as a marker of total linear growth, encompassing the effects of both genotype and environmental factors impacting growth at any stage of development. We used tibia length as a marker of growth that is especially sensitive to environmental factors in early life (Gunnell et al. 1998, 2003).

We focus on the magnitude of effect and 95% confidence intervals rather than *p*‐values.

## 3 Results

Seventy women completed study visits, the majority of them students attending universities in and around London. However, our analysis here is restricted to the 68 women who received the MRI pelvis scan. Fifty‐one percent reported Indian ethnicity, 11% Pakistani, 11% Bangladeshi and 11% Sri Lankan ethnicity; the remaining 16% reported mixed South Asian ancestry. Two participants misreported their height and weight at recruitment, resulting in a measured BMI range of 17–30 kg/m^2^. One participant reported a gestational age of 34 weeks; all other participants were born at 37–42 weeks' gestation.

Mean (SD) age was 24.0 (2.4) years, range 20–28 years. Table 1 shows the descriptive characteristics of the participants. Mean height was 161.2 (SD 6.6) cm, and mean BMI 22.3 (SD 3.5) kg/m^2^.

**TABLE 1:** Description of anthropometric, metabolic and pelvic variables in the sample.

Trait	*n*	Mean	SD
Weight	68	57.8	9.3
Height (cm)	68	161.2	6.6
Tibia (cm)	68	36.8	2.5
Body mass index (kg/m^2^)	68	22.3	3.5
Head girth (cm)	67	53.6	1.4
DXA total lean mass (kg)	68	34.1	4.0
DXA arm lean mass (kg)	68	3.4	0.5
DXA leg lean mass (kg)	68	11.9	1.7
DXA limb muscle mass (kg)	68	15.3	2.2
4‐component model fat mass (kg)	68	20.3	6.7
Kidney mass (kg)	68	0.29	0.05
Spleen mass (kg)	66	0.14	0.05
Heart mass (kg)	67	0.53	0.09
Liver mass (kg)	68	1.21	0.22
Brain mass (kg)	68	1.08	0.08
Bi‐acetabular breadth (cm)	68	13.3	0.7
Bi‐iliac breadth (cm)	68	25.1	1.7
Inlet AP diameter (cm)	67	12.9	0.9
Outlet AP diameter (cm)	58	8.8	0.9
Inter‐spinous diameter (cm)	66	10.9	0.7
Inter‐tuberous diameter (cm)	67	13.3	0.9
Resting energy expenditure (kcal/d)	66	1335	187

Abbreviations: AP, anteroposterior; DXA, dual‐energy X‐ray absorptiometry.

For the PCA analysis of organ and muscle mass, the first score (PCA_organs_) explained 55.2% of the total variation and indexed variation in all the non‐brain organs and muscle mass. The second score (PCA_brain_) explained an additional 17.0% of the variation and indexed variability in brain mass (Figure S1). For the PCA analysis of the pelvic dimensions, the first PCA score (PCA_Pelvis1_) explained 56.7% of the variation and indexed variability in the bi‐iliac, bi‐acetabular and inlet dimensions. The second score (PCA_Pelvis2_) explained an additional 23.0% of the variation and captured variability in the inter‐tuberous and inter‐spinous dimensions (Figure S2). Due to missing measurements, the PCA_organs_ and PCA_brain_ scores were available for 65 participants, while the two PCA_pelvis_ scores were also available for 65 women. However, only 62 participants had valid data from both sets of PCA scores. We used all available data for each analysis, and the relevant sample sizes are given in Table S1, ranging from 62 to 67.

**FIGURE 2:**
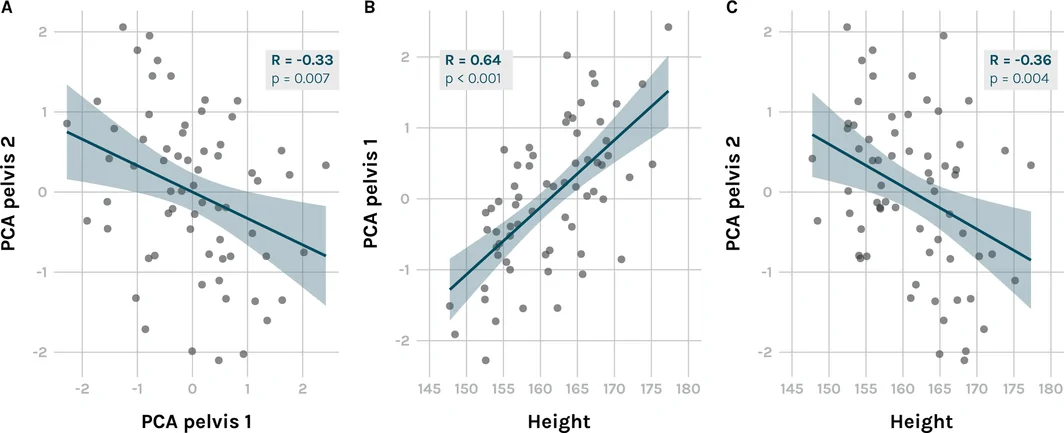
Scatter plots of PC_pelvis1_ with PC_pelvis2_, and of height with PC_pelvis1_ and PC_pelvis2_. The two pelvic components show an inverse association, and while taller height is associated with increased PC_pelvis1_ values, it is associated with reduced PC_pelvis2_ values.

Height was positively correlated with resting energy expenditure, head girth, PCA_organs_ PCA_brain_ and PCA_pelvis1_, and negatively correlated with PCA_pelvis2_ (Table 2). Tibia length was positively correlated with resting energy expenditure, PCA_organs_, PCA_brain_, PCA_pelvis1_ and head girth (though the CI overlapped zero), and negatively correlated with PCA_pelvis2_, though all of these correlations had lower magnitude compared to those for height. Neither height nor tibia length was correlated with fat mass (coefficient close to zero, CI spanning zero). Figure 2 shows scatter plots of the inverse correlation between PCA_pelvis1_ and PCA_pelvis2_, and the contrasting correlations of height with PCA_pelvis1_ and PCA_pelvis2_. Taller height was associated with increased PCA_pelvis1_ values, but with lower PCA_pelvis2_ values.

**TABLE 2:** Correlations of growth markers with metabolic and pelvic traits.

	Tibia length	Height
PCA_organs_	0.46 (0.25, 0.64)	0.53 (0.32, 0.68)
PCA_brain_	0.35 (0.11, 0.54)	0.40 (0.17, 0.59)
PCA_pelvis1_	0.45 (0.24, 0.63)	0.64 (0.47, 0.76)
PCA_pelvis2_	−0.30 (−0.51, −0.06)	−0.36 (−0.55, −0.12)
Resting energy expenditure	0.36 (0.13, 0.55)	0.46 (0.24, 0.63)
Head girth	0.18 (−0.06, 0.40)	0.27 (0.03, 0.48)
Fat mass	−0.02 (−0.26, 0.22)	0.08 (−0.16, 0.31)

*Note:* Values are Pearson correlation coefficients and 95% confidence intervals.

**FIGURE 3:**
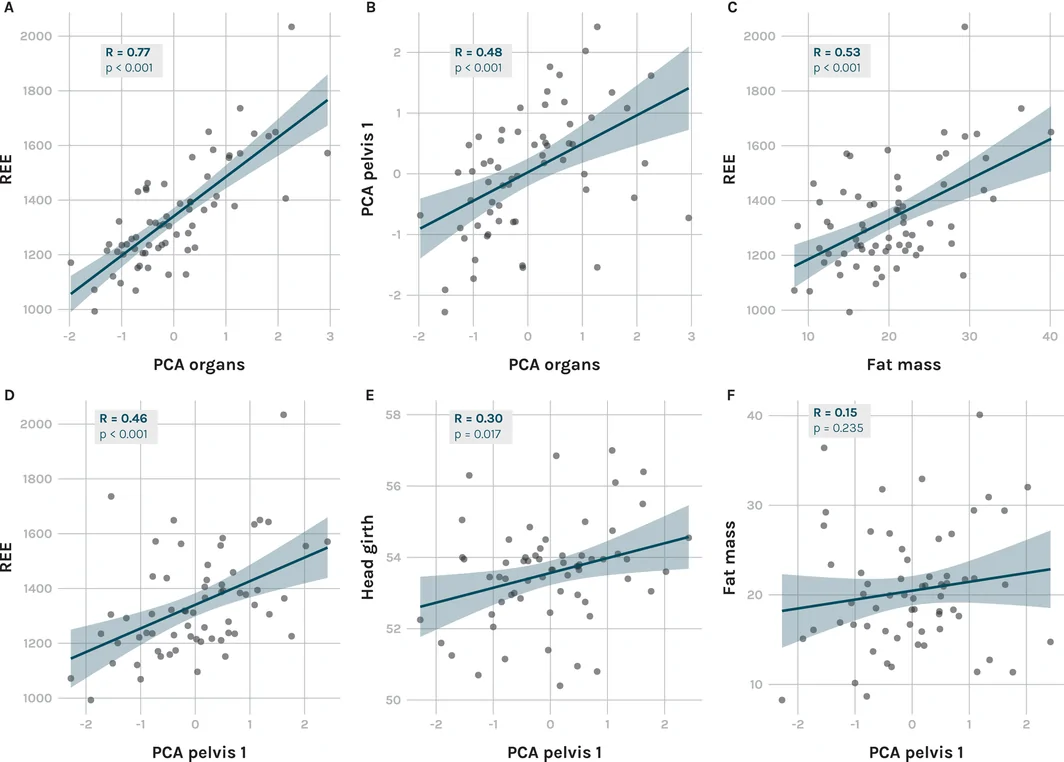
Scatter plots of associations between PC_organs_, PC_pelvis1_, resting energy expenditure (REE), head girth and adiposity. PC_organs_ is positively correlated with both REE and PC_pelvis1_. PC_pelvis1_ is also correlated with REE and head girth. However, fat mass is correlated with REE but not with PC_pelvis1_.

Figure 3 shows scatter plots of selected associations between PCA_organs_, PC_pelvis1_, resting energy expenditure, head girth and adiposity. PCA_organs_ was positively associated with both resting energy expenditure and PCA_pelvis1_, while PCA_pelvis1_ was also positively correlated with resting energy expenditure and with head girth. Fat mass was positively correlated with resting energy expenditure but was not associated with PCA_pelvis1_.

**FIGURE 4:**
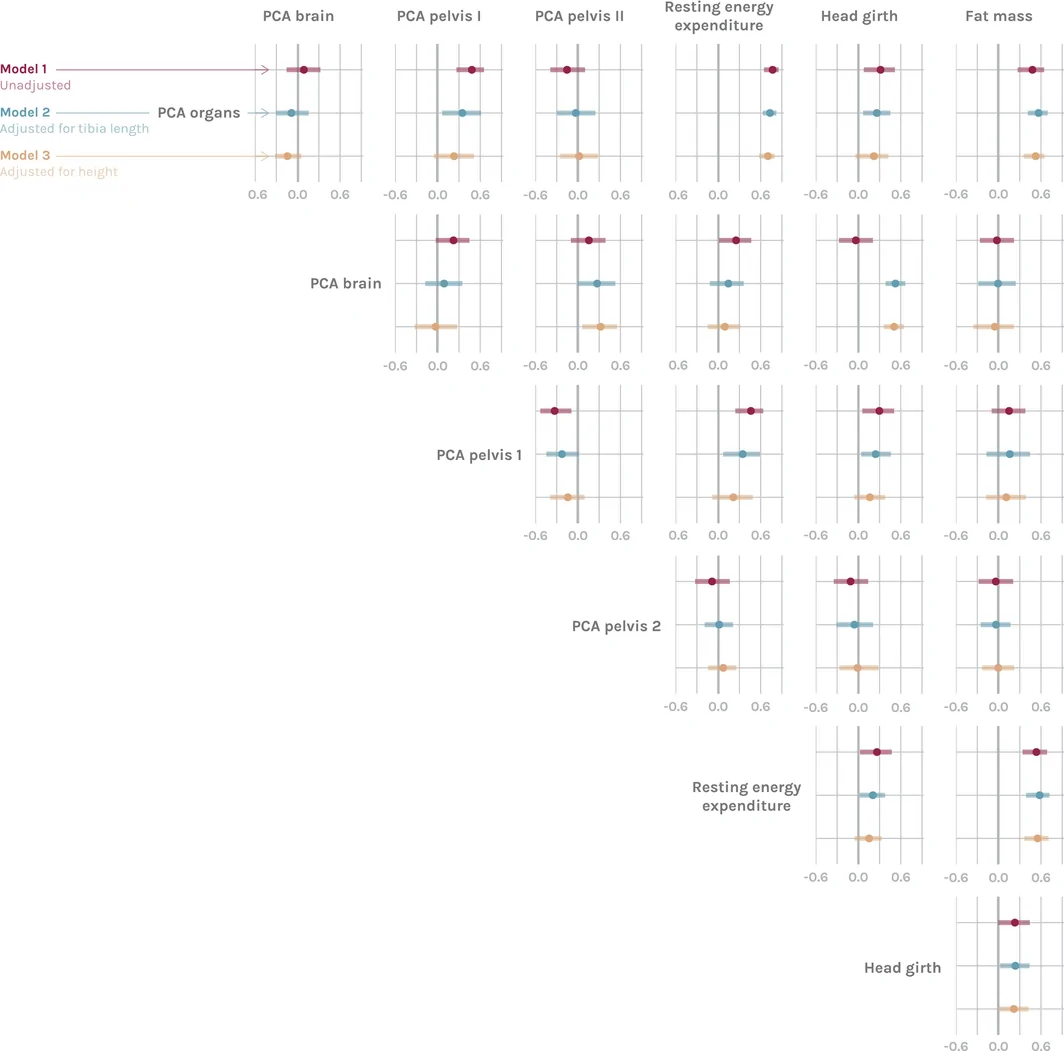
Associations between PC_organs_, PC_brain_, PC_pelvis1_, PC_pelvis2_, resting energy expenditure (REE), head girth and fat mass. Model 1 presents crude associations, Model 2 adjusts for tibia length, and Model 3 adjusts for height. The associations between PC_organs_, PC_pelvis1_ and head girth are partly explained by variability in tibia length and height. The association of PC_brain_ with PC_pelvis1_ is entirely explained by growth variability, whereas the correlation of PC_brain_ with PC_pelvis2_ increases in magnitude when growth is taken into account. The negative association of PC_pelvis1_ and PC_pelvis2_ is largely explained by growth variability.

Figure 4 shows the comprehensive panel of correlations, and how they are affected by adjustment for tibia length and total height (numeric values are given in Supplementary Tables). Positive associations of PCA_organs_ with PCA_pelvis1_, and of PCA_pelvis1_ with resting energy expenditure, were substantially attenuated after adjusting for size, as were the associations of resting energy expenditure and PCA_pelvis1_ with head girth, and resting energy expenditure with PCA_brain_. The inverse association of PCA_pelvis1_ and PCA_pelvis2_ was also attenuated after adjusting for size. Conversely, positive associations of PCA_organs_ with REE, head girth and fat mass, and of resting energy expenditure with fat mass, were little reduced following adjustment for size.

## 4 Discussion

This analysis showed, as hypothesized, that the size of the ‘metabolic core’ and its output of resting energy expenditure were correlated with some dimensions of the obstetric pelvis, including the pelvic inlet. In addition, we replicated associations previously reported between women's pelvic dimensions and their head size (Fischer and Mitteroecker 2015; Greulich et al. 1939). These correlations indicate a degree of coordination of maternal physiological and anatomical traits that respectively contribute to fetal growth and the capacity for childbirth: women metabolically capable of gestating larger offspring also had greater capacity to deliver them. Conversely, although both the metabolic core and resting energy expenditure also correlated with body fat, the latter showed negligible correlation with pelvic dimensions.

Although our data are cross‐sectional, our analyzes indicate that pre‐adult growth is a key underlying driver of the metabolism‐pelvis correlations. From a broad perspective, every metabolic trait that we analyze increases in size through development, hence a common growth drive may contribute to the co‐variation of anatomical and physiological traits observed in adulthood. Beyond that, however, adult organ masses have been shown to correlate with BMI as well as stature (de la Grandmaison et al. 2001), while pelvic dimensions reach their final size after linear growth has ceased (Moerman 1982; Sharma et al. 2016). The two sexes show divergent growth of the pelvis during adolescence, implicating the hormone estrogen (Huseynov et al. 2016). Thus, while overall growth trajectory may be an important factor contributing to correlations of metabolic traits and pelvic dimensions, other factors such as genotype, sex hormones or developmental variability may also contribute. For fat mass, these other factors appear to be the dominant influence, as growth markers explained negligible variability.

As skeletal growth incorporates both genetic and environmental influences, we investigated two different markers. Total stature has relatively high heritability, with coefficients for women ranging from 0.68 to 0.84 depending on the population (Silventoinen et al. 2003). The length of the tibia is considered more sensitive to environmental stressors in early life (Gunnell et al. 1998; Pomeroy et al. 2012), though a study of heritability in 3782 European female twin pairs still found a relatively high heritability coefficient of 0.65. Our analyzes found that correlations of PC_pelvis1_ with PCA_organs_, resting energy expenditure and head girth were all partly explained by growth variability, including tibia variability. Overall, this shows how different periods and components of growth coordinate the metabolic core, resting energy expenditure and pelvic dimensions, with favorable implications for childbirth. Conversely, associations of PCA_organs_ with resting energy expenditure and fat mass were unexplained by growth, indicating that they may simply reflect variability manifesting in adulthood.

Neither PC_pelvis1_ nor PC_pelvis2_ was correlated with fat mass, however whether this has implications for childbirth may depend on other maternal traits. A number of studies from both high‐ and low‐income settings, reviewed previously (Wells et al. 2018; Lederman et al. 1999), have shown that within the normal range of BMI, maternal FFM is a robust predictor of offspring birthweight, whereas maternal adiposity is either a weak predictor or unrelated to birthweight. Accordingly, maternal adiposity may vary to some extent without challenging childbirth. However, at higher levels, maternal adiposity is associated with increased neonatal dimensions and fatness (Wells 2017), mediated by higher levels of fetal insulin (Hapo Study Cooperative Research Group 2009; Anam et al. 2022). Consistent with its contrasting associations with the capacity to promote fetal growth (positive) and with pelvic dimensions (null), high maternal adiposity is an established predictor of C‐section and obstructed labour (Wells 2017; Wells et al. 2018). However, the point at which adiposity is sufficiently high to disrupt fetal‐maternal coordination may also depend on maternal growth and pelvic dimensions.

A number of studies have linked short stature with both smaller pelvic dimensions (Shirley et al. 2020; Adadevoh et al. 1989; Bernard 1950) and an increased risk of childbirth complications, including emergency C‐section and obstructed labour (Wells 2017; Sokal et al. 1991; Khunpradit et al. 2005; Amoa et al. 1997). In addition, as BMI increases, the risk of C‐section increases more in shorter women than in those of taller height (Wells et al. 2018, 2020). Notably, in a chronically undernourished population from rural lowland Nepal (average stature 150.5 cm), even relatively low levels of BMI (> 21 kg/m^2^) were associated with increased risk of obstructed labour by self‐report, compared to women with BMI < 21 kg/m^2^ (Wells, Marphatia, et al. 2021). This suggests that in ancestral populations, fat stored during pregnancy was used to fund lactation, and did not contribute to cumulative BMI increases with age during the reproductive career.

However, both in this and other populations, there are indications of pelvic adaptations to the selective pressure of obstructed labour. In shorter compared to taller women, the pelvis appears to differ not only in size but also in shape. In our previous analysis of this cohort, we reported positive associations of height and tibia length with all 6 pelvic dimensions, though the associations were weaker for inter‐tuberous and inter‐spinous diameters (Shirley et al. 2020). In our current analyzes, however, inter‐tuberous and inter‐spinous diameters showed a pattern of variation (PCA_pelvis2_) that was distinct from the variability in bi‐iliac, bi‐acetabular and inlet dimensions (PCA_pelvis1_). While PCA_pelvis1_ was positively correlated with height, PCA_pelvis2_ showed a negative association. This suggests that the component of variability in inter‐tuberous and inter‐spinous diameters that is distinct from PCA_pelvis1_ helps relieve the effect of shorter stature on PCA_pelvis1_. In other words, taller women tended to have higher PCA_pelvis1_ and lower PCA_pelvis2_, while shorter women tended to have the reverse pattern. The fact that the correlation between PCA_brain_ and PCA_pelvis2_ also increased after adjusting for tibia and more so adjusting for height further suggests that variability in this second component of pelvic variation may help resolve the potential challenges of short stature for childbirth. Similarly, we note that in a Southern African population from the Late Stone Age, female short stature was associated with relatively wide birth canals, indicating a pelvic adaptation to facilitate childbirth (Kurki 2007). In combination, these findings indicate the strength of childbirth as a selective pressure on female pelvic characteristics; nevertheless the gracile physique of South Asian women appears to be associated with relatively smaller absolute pelvic dimensions (Wells et al. 2016).

Nevertheless, such compensation may be only partial, given the tendency for shorter women to experience greater risk of childbirth complications (Wells 2017; Merchant et al. 2001). We have shown previously that both maternal BMI and the rate of caesareans increased between 2005–2016 and 2015–2016 in India, and that around a fifth of the secular increase in caesarean rate is explained by the secular increase in maternal BMI, whereas maternal height shows negligible secular increase (Wells et al. 2018).

Overall, the coordination of metabolic and pelvic traits is expected to ameliorate the obstetric dilemma at the individual level. Women who experience better growth in early life (potentially through both genetic mechanisms and phenotypic plasticity) gain greater capacity to both gestate and deliver the fetus; women with poorer growth sacrifice both capacities, predicted to reduce the risk of childbirth complications. These associations have implications for secular trends in body size. Secular increases in female height could potentially increase not only core metabolic tissues (FFM) but also pelvic dimensions, as reported in the UK (Holland et al. 1982). Conversely, the long‐term downward secular trend in height in India appears to be driven smaller pelvic dimensions, and viable delivery is achieved by a trade‐off with lower birth weight (Wells et al. 2016).

Our study had some limitations. The sample size was relatively small in epidemiological terms, and challenges in extracting MRI outcomes resulted in some missing data, in particular for the pelvic outlet which was therefore excluded from the analysis. The study was performed on nulliparous women with no plans for follow‐up, hence any childbirth outcomes remain unknown, similar to other studies of coordination between the obstetric pelvis and other anatomical traits (Fischer and Mitteroecker 2015). We note that first‐time mothers show increased risk of childbirth complications in many (Wells, Marphatia, et al. 2021; Kabakyenga et al. 2011; Ali and Adam 2010) but not all settings (Desta et al. 2022), which may relate to the lengthy developmental period over which pelvic dimensions reach their maximal values (Sharma et al. 2016). Ideally, we would have been able to follow up these women as they delivered their first offspring, and to have tested our prediction that high adiposity, but not high fat‐free mass, would be associated with the risk of childbirth complications.

The study also focused on women from South Asia, with reduced stature and fat‐free mass compared to the global average (NCD Risk Factor Collaboration 2016; Pomeroy et al. 2019), hence our findings require confirmation in other populations. Ancestry was obtained by self‐report, extending back to the grandparent generation. Assessed in this way, ancestry may reflect both the South Asian gene pool and also the exposure of the women themselves, or their recent ancestors, to South Asian geographical environments. Among the strengths of the study are the high‐quality MRI data on both vital organs and pelvic dimensions, as well as supporting data on limb muscle mass and whole‐body fat mass, and resting metabolic rate in well‐controlled conditions. The exclusion and inclusion criteria controlled for several sources of variability, but also reduce the generalisability of the results.

In conclusion, our analysis found that the metabolic traits that would promote fetal growth during pregnancy, and hence size of the offspring at birth, were correlated with larger pelvic traits and hence the size of the birth canal, due in part to common associations of these traits with variability in linear growth. Variation in growth in early life thus promotes a degree of coordination in women's capacity to both grow and deliver a fetus. However, body fat does not fit this pattern, helping understand why high levels of fat, which may influence fetal growth, can increase the risk of childbirth complications.

## Author Contributions

**Jonathan C. K. Wells:** conceptualization, writing – original draft, methodology, formal analysis, project administration. **Rasmus Wibaek:** writing – review and editing, visualization. **Meghan Shirley Bezerra:** conceptualization, investigation, funding acquisition, writing – review and editing, project administration, data curation. **Owen J. Arthurs:** investigation, writing – review and editing, methodology. **Simon Eaton:** investigation, methodology, formal analysis. **Kiran K. Seunarine:** investigation, methodology. **Chris A. Clark:** conceptualization, investigation, funding acquisition, methodology, supervision.

## Funding

This work was supported by Wenner‐Gren Foundation (8888), National Institute for Health Research (NIHR‐CS‐012‐002), National Institute for Health Research (NIHR) Great Ormond Street Hospital Biomedical Research Centre.

## Conflicts of Interest

The authors declare no conflicts of interest.

## Supporting information


**Table S1:** Sample sizes available for analysis.
**Figure S1:** Principal Component Analysis of organ and muscle mass.
**Figure S2:** Principal Component Analysis of pelvic dimensions.

## Data Availability

The data that support the findings of this study are available on request from the corresponding author. The data are not publicly available due to ethical restrictions relating to the UK General Data Protection Regulation, which determined the wording of the consent forms signed by the participants.
