# Supplementary material for: Organ Size and Metabolic Rate Co‐Vary with Dimensions of the Obstetric Pelvis: The Metabolic Coordination of Childbirth Hypothesis
Source: Am J Biol Anthropol. 2026 Sep 3;191(1):e70342. doi: 10.1002/ajpa.70342 (PMC13541417; doi:10.1002/ajpa.70342)

Organ size and metabolic rate co-vary with dimensions of the obstetric pelvis: the metabolic coordination of childbirth hypothesis

Wells et al.

Supplementary online material

Results of Principal Components Analysis of organ/muscle and pelvic variability

Supplementary Table S1. Sample sizes available for analysis

	PCA organs and brain	PCA pelvis 1 and 2	Head girth	REE
PCA pelvis 1 and 2	62			
Head girth	64	64		
REE	63	63	65	
Fat mass	65	65	67	66

Figure S1: Principal Component Analysis of organ and muscle mass

Kidney, Spleen, Liver, heart, Brain, Limb muscle

Total Variance Explained

Component	Initial Eigenvalues			Extraction Sums of Squared Loadings			Rotation Sums of Squared Loadings[a]
	Total	% of Variance	Cumulative %	Total	% of Variance	Cumulative %	Total
1	3.311	55.187	55.187	3.311	55.187	55.187	3.300
2	1.022	17.040	72.228	1.022	17.040	72.228	1.128
3	.618	10.299	82.526				
4	.469	7.823	90.350				
5	.315	5.250	95.600				
6	.264	4.400	100.000				

Extraction Method: Principal Component Analysis.

a. When components are correlated, sums of squared loadings cannot be added to obtain a total variance.

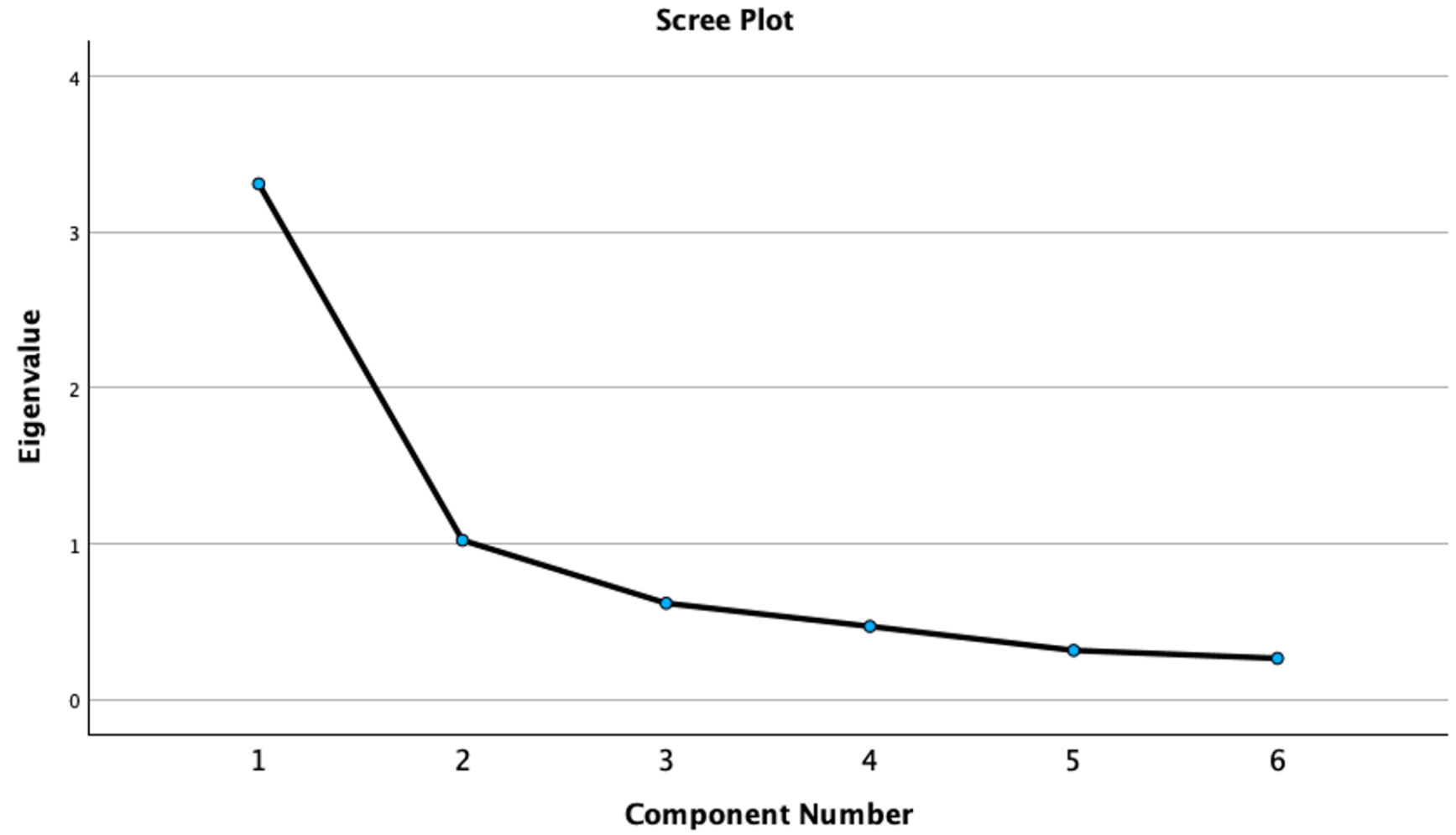

KMO and Bartlett's Test

Kaiser-Meyer-Olkin Measure of Sampling Adequacy.		.848
Bartlett's Test of Sphericity	Approx. Chi-Square	153.230
	df	15
	Sig.	<.001

Pattern Matrix[a]

	Component	
	1	2
Kidney_mass	.771	.168
spleen_mass	.715	-.315
heart_mass	.844	-.092
liver_mass	.844	.080
totalbrainmass_kg	.036	.952
LIMB_Muscle	.863	.089

Extraction Method: Principal Component Analysis.
Rotation Method: Oblimin without Kaiser Normalization.

a. Rotation converged in 3 iterations.

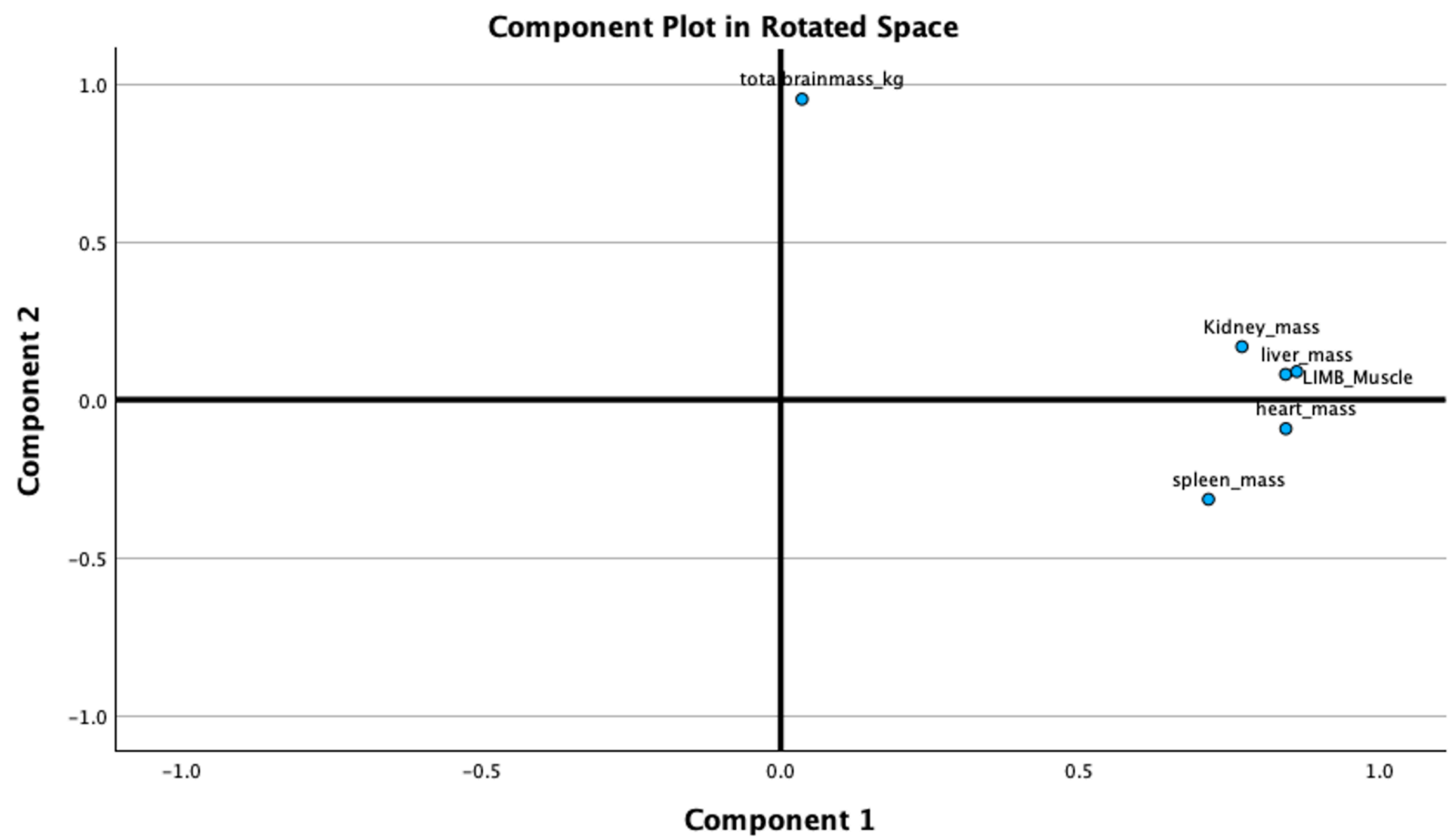

Figure S2: Principal Component Analysis of pelvic dimensions

Bi-iliac, Bi-acetabular, Inlet, Inter-spinous, Inter-tuberous

Total Variance Explained

Component	Initial Eigenvalues			Extraction Sums of Squared Loadings			Rotation Sums of Squared Loadings[a]
	Total	% of Variance	Cumulative %	Total	% of Variance	Cumulative %	Total
1	2.834	56.675	56.675	2.834	56.675	56.675	2.341
2	1.152	23.034	79.709	1.152	23.034	79.709	2.243
3	.598	11.956	91.665				
4	.255	5.097	96.762				
5	.162	3.238	100.000				

Extraction Method: Principal Component Analysis.

a. When components are correlated, sums of squared loadings cannot be added to obtain a total variance.

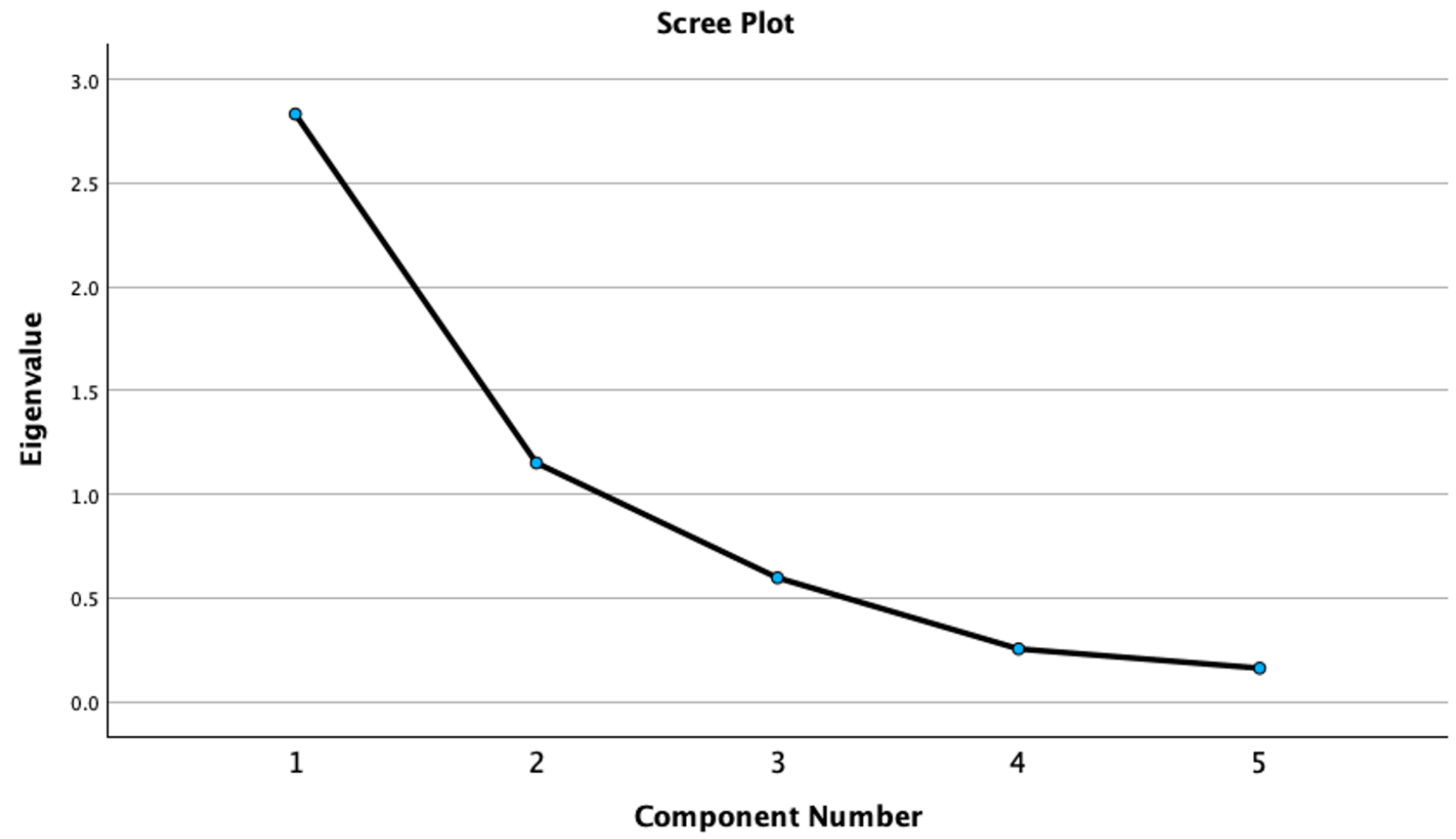

KMO and Bartlett's Test

Kaiser-Meyer-Olkin Measure of Sampling Adequacy.		.644
Bartlett's Test of Sphericity	Approx. Chi-Square	154.947
	df	10
	Sig.	<.001

Pattern Matrix[a]

	Component 1	Component 2
Bi_acetabular	.719	-.336
Biliac	.855	-.059
Inlet	.859	.201
Interspinuous	-.049	-.969
Intertuburous	.084	-.888

Extraction Method: Principal Component Analysis.
Rotation Method: Oblimin without Kaiser Normalization.

a. Rotation converged in 8 iterations.

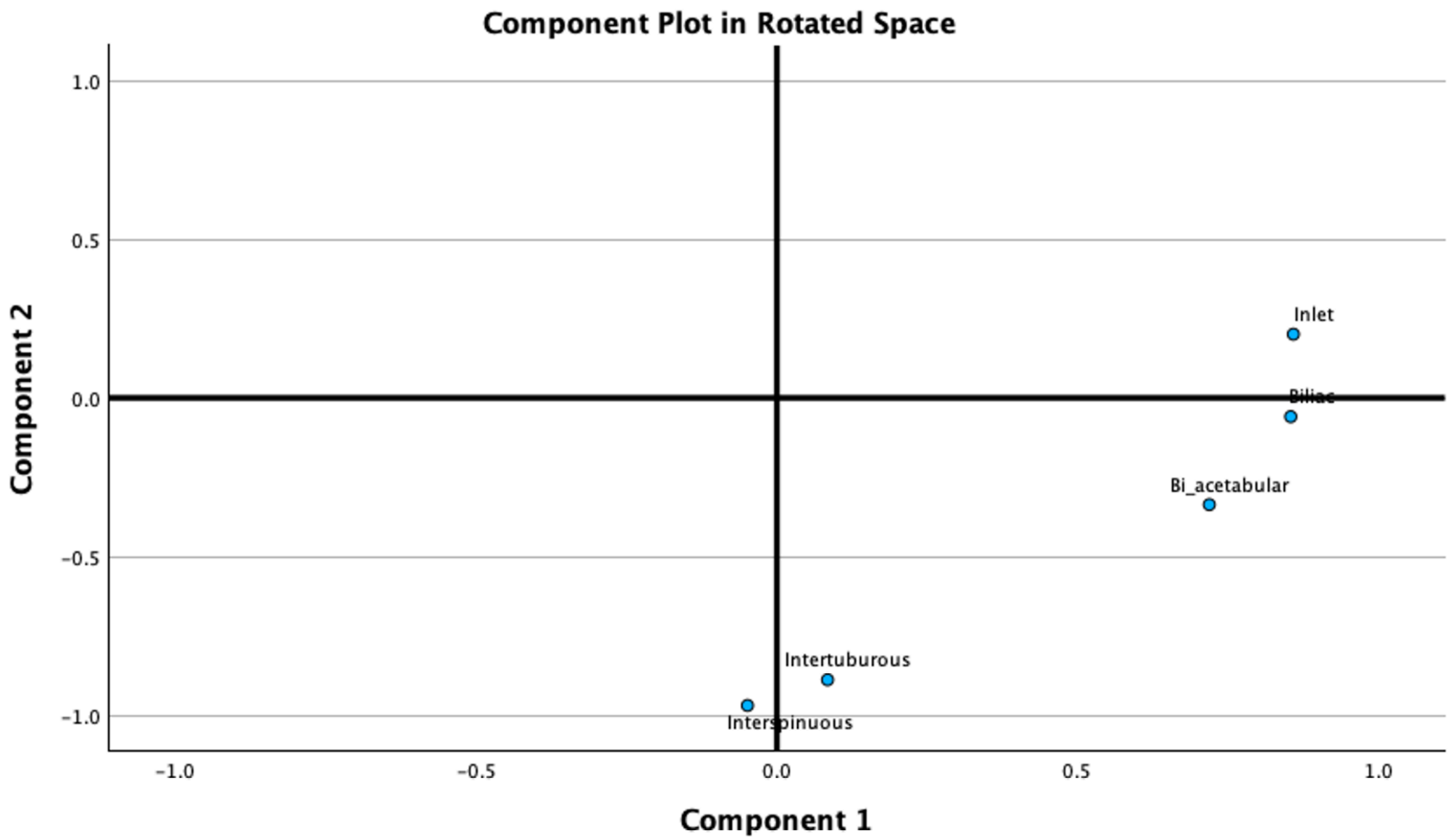

Supplement: Supplementary file 1 [file AJPA-191-e70342-s001.pdf]
